# Regulation and Molecular Basis of Environmental Muropeptide Uptake and Utilization in Fastidious Oral Anaerobe *Tannerella forsythia*

**DOI:** 10.3389/fmicb.2017.00648

**Published:** 2017-04-12

**Authors:** Angela Ruscitto, Kiyonobu Honma, Vamsee M. Veeramachineni, Kiyoshi Nishikawa, Graham P. Stafford, Ashu Sharma

**Affiliations:** ^1^Department of Oral Biology, University at Buffalo, BuffaloNY, USA; ^2^Department of Microbiology and Removable Prosthodontics, School of Dentistry, Aichi Gakuin UniversityNagoya, Japan; ^3^School of Clinical Dentistry, University of SheffieldSheffield, UK

**Keywords:** muropeptides, peptidoglycan, *Tannerella forsythia*, AmpG, GppX

## Abstract

*Tannerella forsythia* is a Gram-negative oral anaerobe associated with periodontitis. This bacterium is auxotrophic for the peptidoglycan amino sugar *N*-acetylmuramic (MurNAc) and likely relies on scavenging peptidoglycan fragments (muropeptides) released by cohabiting bacteria during their cell wall recycling. Many Gram-negative bacteria utilize an inner membrane permease, AmpG, to transport peptidoglycan fragments into their cytoplasm. In the *T. forsythia* genome, the Tanf_08365 ORF has been identified as a homolog of AmpG permease. In order to confirm the functionality of Tanf_08365, a reporter system in an *Escherichia coli* host was generated that could detect AmpG-dependent accumulation of cytosolic muropeptides via a muropeptide-inducible β-lactamase reporter gene. *In trans* complementation of this reporter strain with a Tanf_08365 containing plasmid caused significant induction of β-lactamase activity compared to that with an empty plasmid control. These data indicated that Tanf_08365 acted as a functional muropeptide permease causing accumulation of muropeptides in *E. coli* and thus suggested that it is a permease involved in muropeptide scavenging in *T. forsythia*. Furthermore, we showed that the promoter regulating the expression of Tanf_08365 was activated significantly by a hybrid two-component system regulatory protein, GppX. We also showed that compared to the parental *T. forsythia* strain a mutant lacking GppX in which the expression of AmpG was reduced significantly attenuated in utilizing free muropeptides. In summary, we have uncovered the mechanism by which this nutritionally fastidious microbe accesses released muropeptides in its environment, opening up the possibility of targeting this activity to reduce its numbers in periodontitis patients with potential benefits in the treatment of disease.

## Introduction

*Tannerella forsythia* is a Gram-negative bacterium strongly associated with severe forms of periodontal disease (Socransky et al., 1998; Tanner and Izard, 2006; Kassebaum et al., 2014), a common inflammatory disease worldwide that affects the soft and hard tissues leading to tooth loss (Kassebaum et al., 2014). The dependence of *T. forsythia* on exogenous growth factors becamefirst evident in the studies of Tanner et al. (1986) who noted that this bacterium grew on plates only when it was co-streaked with *Fusobacterium nucleatum*. Subsequently, it was determined that *T. forsythia* is unable to synthesis its own peptidoglycan precursors and has an absolute requirement for the cell wall constituent *N*-acetylmuramic acid (MurNAc) as a growth factor (Wyss, 1989). The dependence of the bacterium on exogenous peptidoglycan precursors became clear when it was observed by Wyss (1989) that the growth of bacterium could be rescued in the presence of *F. nucleatum* co-culture separated by a dialysis membrane. *In vivo, T. forsythia* at least in part, relies on peptidoglycan fragments released by the cohabiting bacteria during their cell wall recycling in the oral cavity. In Gram-negative bacteria, AmpG-like permeases play an important role in the transport of peptidoglycan (muropeptide) fragments from the periplasm to the cytoplasm, which are then broken down further by AmpD (amidase) and processed intracellularly via a salvage pathway and reenter the peptidoglycan synthesis pathway (Park and Uehara, 2008; Reith and Mayer, 2011; Johnson et al., 2013). AmpG belongs to the major facilitator superfamily (MFS) requiring an active proton motive force to transport GlcNAc-anhMurNAc disaccharide and disaccharide carrying stem peptides, the primary products of the action of lytic glycosylases on peptidoglycan, across the inner membrane of these bacteria (Cheng and Park, 2002). However, little is known of the mechanisms by which *T. forsythia* is able to utilize peptidoglycan from the environment. During bioinformatics screening, we identified a candidate gene in the bacterial genome coding for a putative muropeptide permease AmpG. Interestingly, this gene (*Tanf_08365*) is located on an operon that also codes for enzymes that in other bacteria are involved in peptidoglycan recycling as well as protein glycosylation. Interestingly, a recent study reported that this operon, which includes the putative *ampG* gene Tanf_08365 is highly downregulated in a *T. forsythia* mutant deficient in the regulatory protein GppX (Niwa et al., 2011). GppX is a unique hybrid two-component system (TCS) regulator comprising of an N-terminal histidine kinase (HK) sensor domain fused to a central receiver and C-terminal response regulator (RR) domain with a putative AraC-like helix-turn-helix DNA binding motif (Niwa et al., 2011). GppX deletion in *T. forsythia* was shown to pleiotropically affect a range of proteins including the S-layer glycoproteins involved in the virulence of this organism (Niwa et al., 2011). TCSs are signal transduction systems which are comprised of a membrane bound sensor histidine kinase (HK) and a cognate RR (Stock et al., 2000). Upon sensing external stimulus, the sensor kinase autophosphorylates at specific histidine residue and phosphoryl group is then transferred to the cognate RR, and usually enhancing its DNA binding and transcriptional activity (Stock et al., 2000).

Here, we reveal for the first time that Tanf_08365 is a functional AmpG ortholog in *T. forsythia* that is involved in muropeptide transport and furthermore uncover that its regulation is mediated by the direct interaction of GppX with the promoter region of a large operon involved in muropeptide and MurNAc scavenging.

## Materials and Methods

### Bacterial Strains and Growth Conditions

*Escherichia coli* strains (Supplementary Table S1) used in this study were grown aerobically at 37°C in Luria–Bertani broth (LB) medium. All cloning experiments were performed using the electrocompetent recA mutant strain *E. coli* Stellar (Clontech). *T. forsythia* ATCC 43037 wild-type and Δ*gppX* (Niwa et al., 2011) mutant strains were grown anaerobically (10% CO_2_, 10% H_2_, 80% N_2_) in Trypticase Soy Broth (TSB).

### *E. coli* Manipulation by P1 Transduction

*Escherichia coli ΔampG/ampD* double mutant named AR74 was generated by transduction of *E. coli* ampD mutant TU278 with a P1 lysate from an *E. coli* ΔampG mutant JW0423-1. *E. coli* ampD mutant was inoculated in 5 mL LB broth overnight at 37°C. The 1.5 mL of the cell culture was centrifuged at 12, 000 × *g* for 2 min. The supernatant was discarded and re-suspended in one half the original culture volume in sterile P1 solution (10 mM CaCl_2_/5 mM MgSO_4_). One hundred microliters of cell suspension was mixed with varying amounts of lysate (1, 10, 100 μL), incubated for 30 min and then 1 mL of LB broth with 1 M sodium citrate was added. After 1 h incubation at 37°C with gentle shaking, cells were plated on kanamycin plates with 5 mM sodium citrate. The transformed bacteria colonies were selected from the kanamycin plates and were analyzed by PCR to confirm the deletion of *ampG* and *ampD* genes. Colonies that gave the expected size PCR products with primers flanking *ampG* or *ampD* (AmpG forward, AmpG reverse, AmpD forward, and AmpD reverse) were considered double mutants and used further.

### Construction of Tanf_08365 Expression Vector and Assessment of AmpG Function

*Tanf_08365* open-reading frame (ORF) was amplified from *T. forsythia* 43037 DNA with primers (ampGNde-F/ampGHind-R) and cloned into plasmid pACY-AR1 at *Nde*I and *Hin*dIII restriction sites to generate the plasmid pAC-*Tanf_08635*. pACY-AR1 was derived from pACYC184 by inactivating the plasmid backbone *Hin*dIII site by the Q5 Site-Directed Mutagenesis Kit (New England Biolabs), and then replacing the *tet* gene ORF with a short NdeI-HindIII linker using an inverse PCR strategy (In-Fusion, Clontech). DNA sequencing was performed to confirm correct insertion of the linker. For assessing the functionality of *Tanf_08365, E. coli* Δ*ampG/ampD* mutant AR74 was transformed with pNU305 bearing muropeptide inducible lactamase gene (Lindberg et al., 1987) and pAC-*Tanf_08365*. Controls consisted of *E. coli* AR74/pNU305 with pACY-AR1 empty plasmid and lactamase assays were performed as below.

### β-lactamase Induction and Assay

The assays were performed as described previously (Zhang et al., 2010). *E. coli* strains were grown to an OD_600_ of 0.1 and 1 μg/mL cefoxitin (Sigma) at 42°C was added to induce cell wall disruption. At 0, 30, 60 min post induction, 10 mL bacterial suspensions were taken, centrifuged for 10 min at 6,000 × *g*. Cells extracts obtained by brief sonication and centrifugation at 16, 000 × *g* for 5 min in cold. Lactamase activity of each strain lysate was assayed using the chromogenic nitrocefin substrate (Calbiochem). Briefly, nitrocefin stock solution (500 μg/mL in DMSO) was diluted 10-fold in 0.1 M phosphate, 1 mM EDTA pH 7.0 buffer and 5 μl of diluted solution was then added to 100 μL of cell lysate. After 30 min incubation at 20°C, absorbance was measured at OD_486_ in a microplate reader.

### Reverse Transcription-polymerase Chain Reaction

Total RNA was isolated from bacteria using the RNeasy kit (Qiagen). Single-stranded cDNA was synthesized using reverse transcriptase (Invitrogen Superscript III) and random hexamer primers as per the manufacturer’s protocol. The synthesized cDNA was amplified by PCR with primer sets spanning target genes Tanf_08345-Tanf_08365: region ‘a’ with TF1059F/TF1061R; region ‘b’ with TF1061F/TF1062R; region ‘c’ with TF1062F/TF1063R; region ‘d’ with TF1063F/TF1064R; region ‘e’ with TF1064F/TF1065R, and region ‘f’ with TF1065-TFIF. Primer sequences are listed in Supplementary Table S2.

### 5′ RLM-RACE

5′ RLM RACE was performed to identify the transcription start site with the FirstChoice RLM-RACE kit (Ambion). Briefly, 5′ RACE adapter provided in the kit was ligated to RNA isolated by the Qiagen RNeasy Mini Kit with T4 RNA ligase. Reverse transcription reaction was then performed on the ligated RNA by random priming and Reverse Transcriptase. RT reaction then underwent an Outer 5′ RLM-RACE PCR using primers 5′ Outer Primer (provided by kit) and TSS-Outer. This was followed by a second PCR reaction (Inner 5′ RLM RACE) using primers 5′ Race Inner Primer (provided by kit) and TSS-Inner. PCR products were cloned into pGEM-T (Promega) cloning vector and sequenced.

### Construction of *ampG* Promoter-l*acZ* and *lac-gppX* Chimeras for Assessment of GppX as Transcription Activator

DNA fragment encompassing nucleotides -400 to +1 (transcription start site) of the ampG operon was amplified with primer set ampGProEcoF/ampGProRBam and cloned into plasmid pRS414 into BamH1 and EcoR1 restriction sites to generate recombinant pRS-AmpGpromo. In parallel, a chimeric DNA fragment comprising an IPTG inducible synthetic lac promoter fused in front of a gppX ORF was generated by an overlap PCR strategy. Briefly, a PCR fragment was generated with *T. forsythia* 43037 DNA as a template and primer set LacGppxF1/GppxXho1. The product of this PCR was used as a template in a second PCR with primer set LacGppx-BamF2/GppxXho1). The PCR product was then cloned into plasmid pACY-AR2 into BamH1 and Xho1 to generate pAC-lacTFgppX. In addition, a construct having deletion of C-terminal helix-turn-helix (HTH) domain encoding fragment was derived from pAC-lacTFgppX by inverse PCR, and named pAC-lacTfgppΔHTH,

### β-galactosidase Assay

β-galactosidase activity was determined as described previously (Miller, 1992). *E. coli* strains were inoculated in overnight cultures, diluted 1/100 in 10 mL of fresh medium and induced with isopropyl β-D-1-thiogalactopyranoside (IPTG) until grown to mid-log phase. After mid-log phase growth was reached, cultures were incubated on ice for 20 min and pelleted by centrifuging for 10 min at 6,000 rpm. Cell pellets were re-suspended in the same volume of Z buffer (0.06 M Na_2_HPO_4_⋅7H_2_O, 0.04 M NaH_2_PO_4_⋅H_2_O, 0.01 M KCl, 0.001 M MgSO_4_, and 0.05 M β-mercaptoethanol) and OD_600_ was taken. Cells were diluted in Z buffer to 1 mL, permeabilized by adding 100 μL chloroform and 50 μL of 0.1% SDS, vortexed, and incubated for 5 min at 28°C. Enzyme activity (Miller Units) was measured by adding 0.2 mL of *O*-nitrophenyl-β-D-galactoside (ONPG; 4 mg/mL) substrate in phosphate buffer (0.06 M Na_2_HPO_4_⋅7H_2_O, 0.04 M NaH_2_PO_4_⋅H_2_O) to pH 7.0. OD 420 and 550 after sufficient yellow color has been seen. Add 0.5 mL of 1 M Na_2_CO_3_ to stop the reaction.

### Preparation of Muropeptide Oligomers and Analysis

A previously described protocol (Desmarais et al., 2014) was followed for purifying peptidoglycan from *F. nucleatum* ATCC 25586 cells. *F. nucleatum* was inoculated in 250 mL of BHI broth and grown to OD_600_ of 0.6. Cultures was then spun at 5,000 g for 10 min and re-suspended in 3 mL of phosphate buffered saline (PBS). Cell suspension was added to boiling 6 mL 6% sodium dodecyl sulfate (SDS) solution and continued to boil for 3 h, and then left to stir overnight. Next day ultracentrifugation at 400,000 × *g* for 20 min was continually done until all the SDS was fully removed. The cell wall pellet was then treated with Pronase E (100 μg/mL final concentration) at 60°C for 2 h, ultracentrifuged and treated overnight with muramidase (40 μg/mL final concentration). Muropeptides released after digestion were collected by centrifugation at 15, 000 × *g* for 10 min at room temperature.

Fluorophore-assisted carbohydrate electrophoresis (FACE) was performed to check the quality of the peptidoglycan fragments as described previously (Young, 1996). Briefly, 5–10 μL of isolated peptidoglycan was dried using centrifugal vacuum evaporator and 5 μL of 0.2 M ANTS in 2.6 M acetic acid and 5 μL of 1 M NaCNBH_3_ in DMSO was added. The sample was incubated for 37°C for 15–18 h and vacuum centrifuged overnight. The sample was then separated by electrophoresis using a 35% acrylamide gel (Young, 1996). For muropeptide analysis by mass spectrometry, muropeptide oligomers collected after muramidase treatment were concentrated *in vacuo* to 20 μl and then acidified by addition of 10% formic acid solution. LC-MS analysis was carried out by injecting 7.5 μl of the concentrated muropeptide solution onto a ZORBAX SB-C18 reversed-phase HPLC column (5 μm, 4.6 mm × 150 mm) attached to a Thermo Finnigan LCQ advantage mass spectrometer. The muropeptides were eluted at 200 μl/min using a gradient protocol with 0.1% aqueous formic acid (solvent A) and 0.1% formic acid in acetonitrile (solvent B). The gradient conditions were 0 to 45 min – linear 0 to 20% solvent B; 45 to 90 min – linear 20 to 50% solvent B; 90 to 120 min – linear 50 to 100% solvent B. The resulting LC-MS data was analyzed using Xcalibur QualBrowser program (Thermo-Electron, San Jose, CA, USA).

### Muropeptide Utilization by *T. forsythia*

*Tannerella forsythia* wild-type and Δ*gppX* mutant strains were grown in TSB-serum (TSB medium with 5% fetal bovine serum) supplemented with 0.2% muropeptides or 0.2% MurNAc. TSB-serum medium was used as a negative control. Growth was measured at OD_600_ for 8 days.

### Statistical Analysis

Statistical differences were analyzed by ANOVA, and paired comparisons were performed by Tukey’s *post hoc* test. Statistical analyses were performed with the Prism Software (Graph Pad, San Diego, CA, USA). Data were expressed as mean ± SD and differences were considered to be statistically significant at *P* < 0.05.

## Results

### Tanf_08365 is an AmpG Permease

**FIGURE 1:**
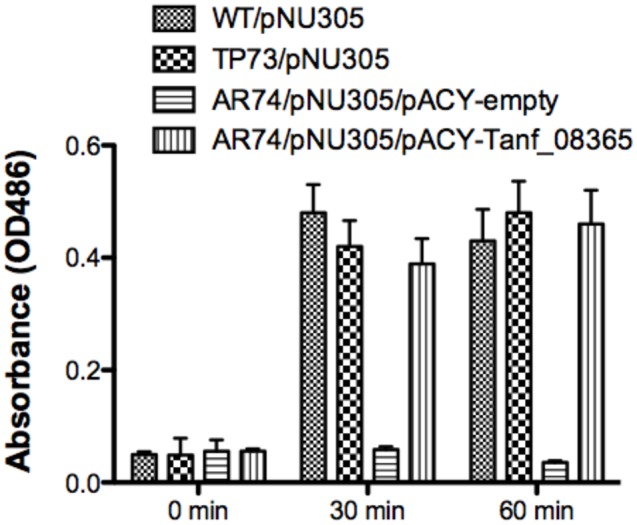
**Reporter assay for detection of AmpG permease activity.** The β-lactamase assay was performed on *Escherichia coli* strains BW25113 (parental strain), TP73 (Δ*ampD*)/pNU305, AR74 (Δ*ampG/*Δ*ampD*)/pNU305/ pACY-AR1 (empty vector), and AR74/pNU305/pAC-Tanf_08635. Activity was determined by chromogenic nitrocefin substrate induced at 0, 30, 60 min and absorbance was measured at OD_486_. Data are representative of three independent experiments with similar results. Each value represents the mean (±SD) of three values measured in one representative assay.

In *E. coli* and many other bacteria, muropeptides are transported across the inner membrane through an AmpG permease (Park and Uehara, 2008). Given the reliance of *T. forsythia* on muropeptide scavenging for survival, we set out to identify an AmpG ortholog in *T. forsythia*. *In silico* analysis of the *T. forsythia* ATCC 43037 draft genome (JUET00000000.1)^1^ indicated Tanf_08365 as a potential MFS protein similar to AmpG permease having 12 transmembrane helices based on a transmembrane helical and topology prediction by the HMMTOP model (Tusnady and Simon, 2001) via the ExPASy server. Tanf_08365 ORF showed 24 and 79% amino acid sequence identity with the AmpG protein of *E. coli* (accession no. WP_021557614.1) and *Bacteroides thetaiotaomicron* (accession no. WP_055269099.1), respectively. To assess the function of Tanf_08365 in muropeptide recycling, an *E. coli* reporter system was generated that relies on the induction of β-lactamase from a reporter plasmid only when a high enough concentration of muropeptides accumulate in the cell via a functional AmpG permease. The reporter *E. coli* strain is a double mutant lacking the *ampG* and *ampD* genes and harbors a plasmid with muropeptide inducible β-lactamase gene. This is enabled by the fact that the native muropeptide uptake gene (*ampG*) is absent while deletion of the murein amidase (*N*-acetyl-anhydromuramyl-L-alanine-amidase) gene *ampD* prevents degradation of muropeptides, enabling accumulation of muropeptides in the cytoplasm. Thus, β-lactamase expression is disabled in this strain unless a functional muropeptide transporter is provided *in trans* to enable muropeptide accumulation in the cytoplasm. Therefore, this reporter system allowed us to test the function of the heterologous *T. forsythia ampG* homolog based on the induction of lactamase activity in the reporter *E. coli*/plasmid strain. To generate this reporter system, an *E. coli* Δ*ampD* mutant was transduced with a P1 lysate prepared from an *E. coli* Δ*ampG* mutant to obtain *E. coli* Δ*ampG/*Δ*ampD* double mutant. This double mutant, named AR74, was then transformed with the pNU305 reporter plasmid expressing β-lactamase from a muropeptide inducible promoter element. A randomly selected positive transformant, named AR74/pNU305, served as the reporter strain. In parallel, the Tet resistance gene of pACYC184 plasmid was replaced with the Tanf_08365 ORF using In-Fusion cloning strategy to generate the recombinant plasmid pAC-Tanf_08635. AR74/pNU305 was transformed with either pAC-Tanf_08635, or the pACY empty vector. *E. coli* wild-type strain BW25113 and *E. coli* Δ*ampD* mutant served as positive controls. The β-lactamase induction at various time intervals was determined using the chromogenic substrate nitrocefin by measurement of product at 486 nm. The results showed that when Tanf_08365 (Tf ampG) was provided *in trans*, the reporter strain expression of β-lactamase was significantly induced in comparison to the empty vector control and the levels were similar to that in the *ampD* single mutant where the *E. coli ampG* gene is functional (**Figure 1**). Together, these data indicated that Tanf_08365 is a functional muropeptide permease AmpG of *T. forsythia*, and we label Tanf_08365 as Tf AmpG.

### Regulation of AmpG Operon

**FIGURE 2:**
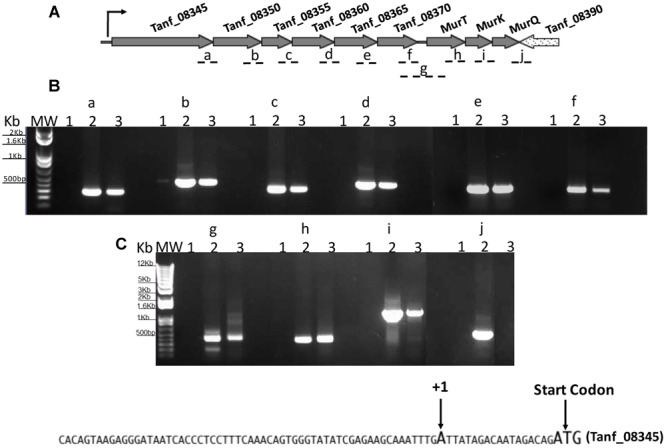
**Analysis of AmpG and MurNAc utilization operon.** RT-PCR analysis with **(A)** primer sets spanning adjacent genes (fragments a-j). Tanf_08345, xanthane lyase; Tanf_08350 (*gtf*), glycosyltransferase; Tanf_08355 (*gtf*), glycosyltransferase; Tanf_08360 (*lytB*), amidase enhancer precursor; Tanf_08365 (*ampG*), muropeptide permease; Tanf_08370 (*ybbC*), conserved hypothetical protein. **(B)** PCR products were separated on a 1% agarose gel. RNA samples with no reverse transcription reaction as template controls were run in lanes 1, genomic DNA as template in lanes 2, and cDNA as template for each primer set in lanes 3. MW; DNA ladder. **(C)** DNA sequence showing transcriptional start site determine by 5′RACE.

**FIGURE 3:**
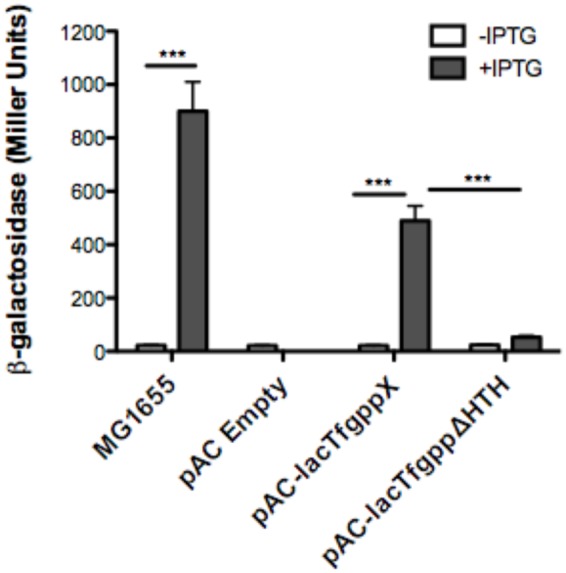
**Reporter assay to assess GppX as a transcription regulator.** The β-galactosidase assay was performed on *E. coli* strains MG1655 (positive control), pAC-lacTFggpΔHTH (negative control), pACYC184 (empty vector), and pAC-lac-gppX (*in trans*). Activity was determined by chromogenic *O*-nitrophenyl-β-D-galactoside (ONPG) substrate with, or without induction of isopropyl β-D-1-thiogalactopyranoside (IPTG) and measured using Miller Units. Each value represents the mean (±SD) of three values measured in one representative assay. Data are representative of three independent experiments with similar results, ^∗∗∗^*P* < 0.001.

We next wanted to ascertain how expression of AmpG might be regulated in *T. forsythia*. The genetic context of Tf AmpG shows that it is potentially part of a large gene cluster in the chromosome stretching from Tanf_08345-Tanf_08370 in the contig_82 DNA sequence (NZ_JUET01000082) (**Figure 2**). Interestingly, the Tanf_08345-Tanf_08370 gene cluster is located immediately upstream of a three gene operon Tanf_08375-Tanf_08385 for MurNAc utilization recently identified by our group (Ruscitto et al., 2016). This operon expresses a transporter TfMurT/TfMurK that transports and phosphorylates environmental MurNAc and an enzyme TfMurQ etherase that converts cytoplasm internalized MurNAc-6-P to GlcNAc-6-P for shuttling into glycolytic pathway or peptidoglycan biosynthetic pathway. Previous work by Niwa et al. (2011) showed that transcription of genes associated with the Tanf_08345-Tanf_08370 cluster [which includes the *ampG* (Tanf_08365) gene studied here] were significantly downregulated in a mutant lacking the GppX regulator protein. To confirm that genes on this cluster indeed form an operon, total RNA from *T. forsythia* ATCC 43037 cells was extracted and co-transcription of the relevant genes was analyzed using RT-PCR as outlined in **Figure 2A**. The results showed that Tanf_08345 to Tanf_08370 are transcribed as a single RNA transcript (**Figure 2B**), since PCR products of the expected size were obtained with primer pairs (Supplementary Table S2) designed to bridge the ends between the ORFs of adjacent genes, and, thus, yielding amplification products only when co-transcription was occurring. In addition, our data showed that the previously identified *murTKQ* gene cluster formed a contiguous transcription unit with the upstream ampG operon, i.e., from Tanf_08345-Tanf_08385. Additionally, our data defined that *murQ* is the final gene in this operon since a primer set bridging the region between *murQ* and *Tanf_08390* yielded no PCR product in these experiments (**Figure 2B**). After establishing that the *T. forsythia ampG gene Tanf_08365* (Tf *ampG*) is part of this operon, the transcription site for the operon was determined; which was located 17 bp upstream (an ‘A’ residue) from the translational start codon of Tanf_08345 (**Figure 2C**). Given that the genes in the cluster are co-transcribed and that the levels of transcription of all the genes in the cluster are downregulated in a gppX mutant (Niwa et al., 2011), we set out to determine whether the regulation by GppX might be direct. In order to test this we constructed a reporter system where the putative promoter region of this operon encompassing -400 to +1 (TSS) region (5′ of the first gene Tanf_08345) was cloned upstream of a promoterless *lacZ* ORF in a reporter plasmid pRS414 and placed in *E. coli* (Simons et al., 1987). In parallel, a synthetic IPTG inducible *lac* promoter/operator fused to *T. forsythia* GppX coding fragment with a C-terminal 6xHis tag sequence was obtained by overlap PCR to generate *lac*-*gppX-6xHis* chimera Tetracycline resistance gene in pACYC184 was then replaced with this chimeric fragment as described in “Materials and Methods” section. In addition, a chimeric fragment lacking HTH domain of GppX with a 6xHis tag (*lac-gppxΔHTH-6xHis)* was also cloned into pACYC184. *T. forsythia* promoter-*lacZ* and *lac-gppX-6His* (*or lac-gppx*Δ*HTH-6xHis*) constructs were placed in the same strain of *E. coli* and the expression of β-galactosidase with and without IPTG induction was determined with chromogenic ONPG. This allowed us to test the hypothesis that GppX might directly regulate the AmpG containing operon mentioned above. The results showed that the operon promoter is induced sevenfold when expression of GppX is turned on with IPTG. In contrast, in the absence of IPTG the GppX construct fails to elicit this response. A strain that contained vector with IPTG inducible Tet gene as control showed no *lacZ* expression (negative control). In addition, a reporter strain containing pAC-lacTfgppΔHTH that lacked the DNA binding HTH domain of GppX showed no *lacZ* expression after induction with IPTG. The western immunoblotting results using anti-His tag antibody showed expected size expressed proteins for each of the chimeric constructs (full length GppX or ΔHTH-GppX) (Supplementary Figure S3); confirming that the reduced promoter induction in the *E. coli* cells expressing ΔHTH GppX construct is not due to reduced protein expression or degradation of the recombinant protein, but is due to its lack of DNA binding ability. *E. coli* strain MG1655 was used as a positive control for lacZ responsiveness as it contains the native IPTG inducible lac operon containing β-galactosidase (**Figure 3**).

### GppX Regulates Muropeptide Dependent Growth

**FIGURE 4:**
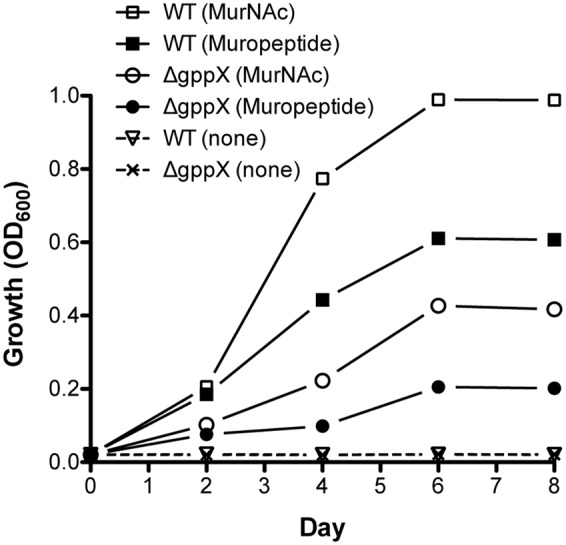
**Growth of *Tannerella forsythia* on muropeptides.** Growth of *T. forsythia* wild-type and ΔgppX in M9 liquid medium supplemented with 0.2% muropeptides or 0.2% MurNAc was measured at OD_600_. Results of one out of three independent cultivations with similar outcome are given.

*Tannerella forsythia* is auxotrophic for peptidoglycan amino sugar MurNAc, and thus requires exogenous MurNAc for growth *in vitro*. Our data so far had highlighted that *T. forsythia* produces a muropeptide transport system encoded by the *ampG* gene that is part of an operon directly under the control of the GppX transcription factor. We therefore surmised that *T. forsythia* should not only be able to grow on muropeptides as growth factors but also that this function should be dependent on GppX. In addition, since the genes involved in MurNAc utilization are part of the same operon we predicted that MurNAc utilization in *T. forsythia* would also be under the control of GppX. As a first step in examining these hypotheses, we set out to examine whether *T. forsythia* can utilize exogenous muropeptides as these would be readily available *in vivo* in the oral cavity as byproducts of cell wall recycling or death of cohabiting bacteria. To test this, muropeptides were prepared from *F. nucleatum* after muramidase digestion as described in the section “Materials and Methods.” The quality and composition of muropeptide fraction was confirmed by subjecting the isolated fraction to FACE and Mass Spectrometry. As shown, a typical ladder-like pattern was observed on a FACE gel (Supplementary Figure S1), and MS analysis (Supplementary Figure S2) confirmed the presence of muropeptide oligomers in the isolated fraction. *T. forsythia* was grown in broth supplemented with muropeptides in the absence of MurNAc supplementation. The data showed for the first time that *T. forsythia* grew in broth supplemented with muropeptides prepared from *F. nucleatum* alone or MurNAc (positive control) (**Figure 4**).

Since previous data had indicated that GppX controlled the *ampG* containing operon we set out to now test whether a GppX mutant was deficient in its ability to grow on these muropeptides in this assay. The results showed that in the presence of muropeptides the growth of the wild-type bacteria was significantly higher than that of the mutant strain; reaching fivefold higher OD_600_ values. Moreover, given that the *murTKQ* genes for MurNAc utilization are part of a contiguous operon controlled by GppX the growth rate in the presence of MurNAc was also predictably lower for GppX-deficient mutant compared to the wild-type strain. Taken together, the reduction in growth displayed by the Δ*gppX* mutant strain demonstrated that GppX via takes an important role in the regulation of peptidoglycan transport.

## Discussion

It was first reported by Tanner et al. (1986) that the growth of *T. forsythia* could be rescued by co-culturing the bacterium with *F. nucleatum*, which was thought to provide peptidoglycan precursors. Subsequently, Wyss (1989) demonstrated that the growth of *T. forsythia* could be rescued in the presence of exogenous peptidoglycan amino sugar MurNAc. The reason for *T. forsythia’s* strict dependence on exogenous MurNAc for growth became evident with the availability of the genome sequence of the organism. The genes for MurA and MurB enzyme homologs involved in the biosynthesis of MurNAc from GlcNAc were found to be absent in the organism (Sharma, 2011). Thus, it is thought that in the hostile oral environment *T. forsythia* salvages peptidoglycan fragments (muropeptides) released during the cell wall recycling of cohabiting bacteria. The peptidoglycan scavenging by *T. forsythia* becomes even more relevant given the fact the human host does not make the peptidoglycan amino sugars. Since the AmpG permease in bacteria plays a major role in the recycling of muropeptides (Cheng and Park, 2002), we sought out to identify a functional homolog of AmpG and its regulation in *T. forsythia*.

This study identified and confirmed that the gene *Tanf_08365* in *T. forsythia* codes for a functional muropeptide permease, which we designate Tf AmpG. Furthermore, we show that AmpG expression in *T. forsythia* is under the direct control of the GppX (Tanf_13760) regulator (Niwa et al., 2011), a hybrid two-component regulatory protein with an N-terminal HK and a C-terminal RR. A previous study reported that deletion of GppX protein Tanf_13760 resulted in the reduced transcription of glycosylation related operon Tanf_08345-Tanf_08370 in *T. forsythia*, of which Tanf_08365, the focus of our study here, is part of (Niwa et al., 2011). However, it could not be ascertained from this previous study whether GppX influences the transcription of this operon directly or indirectly. TCSs are known to impact expression of target genes directly or through mediation of collateral regulatory networks (Graham et al., 2002). Our data presented here utilizing a reporter system in *E. coli* showed that the promoter driving the expression of Tanf_08345-Tanf_08370 operon was directly regulated by GppX in the *E. coli* heterologous system where a direct regulation and interaction with promoter is the only explanation for the data observed. The direct involvement of GppX interaction with the promoter was further corroborated from the results showing that a construct lacking the DNA binding HTH domain of GppX failed to activate the promoter.

**FIGURE 5:**
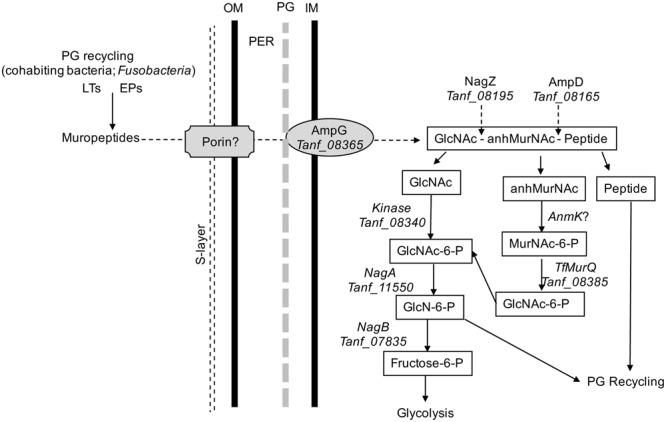
**Schematic model of *T. forsythia* peptidoglycan recycling pathway.** Abbreviations: anhMurNAc, anhydro-*N*-acetylmuramic acid; EPs, endopeptidases; GlcNAc, *N*-acetylglucosamine; GlcNAc-6-P, *N*-acetylglucosamine-6-phosphate; GlcN-6-P, glucosamine-6-phosphate; IM, inner cytoplasmic membrane; LTs, transglycosylases; MurNAc-6-P, *N*-acetylmuramic acid 6-phosphate; OM, outer membrane; PER, periplasmic space.

In addition, in this study we provided direct evidence that *T. forsythia* can utilize exogenous muropeptides and *F. nucleatum* ATCC 25586 can support *T. forsythia* growth. As shown, the growth of *T. forsythia* in muropeptides extracted from *F. nucleatum* revealed that peptidoglycan fragments can sustain *T. forsythia* growth. To validate the role of GppX in the regulation of muropeptide transport via AmpG permease, the growth of *T. forsythia* wild-type and ΔgppX strains were compared. The results showed that the growth of wild-type bacteria was higher than that of the mutant strain. The reduction in growth displayed by the Δ*gppX* mutant strain demonstrated that peptidoglycan transport in *T. forsythia* dependent on AmpG permease is regulated by GppX. Furthermore, since the growth of the mutant was not completely abolished on MurNAc or muropeptides suggest that the *ampG*-containing operon is expressed constitutively at basal levels. These data are line with the previous study showing that *gppX* mutation leads to down regulation but not complete abolition of expression of the operon (Niwa et al., 2011). Although we were not able to complement the Δ*gppX* mutant with a functional GppX protein via a strategy involving replacement of *gppX*::Em locus by a *gppX-Cm* (chloramphenicol marker attached to wild-type *gppX*) fragment, our data from the *E. coli* reporter strain do confirm that GppX protein is involved in the regulation of MurNAc/muropeptide operon. We believe growth attenuation and possibly other changes due to GppX deficiency made bacterial cells less amenable to genetic manipulation. Furthermore, dependence on *F. nucleatum* peptidoglycan fragments by *T. forsythia* is likely responsible for the close physical association in the dental plaque and synergy between these two organisms in terms of virulence. Previous studies have shown that *T. forsythia* and *F. nucleatum* form synergistic biofilms *in vitro* (Sharma et al., 2005), in mixed oral infection stetting induce synergistic alveolar bone loss in a mouse oral infection model (Settem et al., 2012), and are present in close proximity to each other in a human subgingival plaque biofilms (Zijnge et al., 2010). In summary, the nutritionally fastidious periodontal pathogen *T. forsythia* unable to synthesis its own peptidoglycan amino sugar MurNAc scavenges muropeptides from the environment via AmpG-dependent peptidoglycan recycling pathway (**Figure 5**), whose expression is regulated by a hybrid two-component transcription regulator, GppX. We envision a possibility wherein small molecule inhibitors targeting GppX regulator could be developed as antimicrobial compounds against *T. forsythia*, and thus as treatment against periodontitis.

## Author Contributions

Conceived and designed the experiments: AR and AS. Performed the experiments: AR, KH, and VV. Analyzed the data: AR, KH, VV, KN, GS, and AS. Contributed reagents/analysis tools: KN, VV, and GS. Wrote the paper-original: AR, VV, GS, and AS. Review and editing: AR, GS, and AS.

## Conflict of Interest Statement

The authors declare that the research was conducted in the absence of any commercial or financial relationships that could be construed as a potential conflict of interest.

## Footnotes

1 http://www.ncbi.nlm.nih.gov/nuccore/JUET00000000.1
