# Supplementary material for: Regulation and Molecular Basis of Environmental Muropeptide Uptake and Utilization in Fastidious Oral Anaerobe Tannerella forsythia
Source: Front Microbiol. 2017 Apr 12;8:648. doi: 10.3389/fmicb.2017.00648 (PMC5388701; doi:10.3389/fmicb.2017.00648)
Supplement: Supplementary file 1 [file Data_Sheet_1.DOCX]

**Table S1:** *E. coli* Strains and Plasmids used in this study

| *Strain/plasmid* | *Description* | *Reference* |
| --- | --- | --- |
| TU278 | *E. coli* ∆ampD728::frt | Bernhardt lab collection, Harvard Medical School |
| JW0423-1 | *E. coli* ΔampG::kan | Keio collection |
| BW25113 | *E. coli* K-12 | The Coli Genetic Stock Center at Yale |
| MG1655 | *E. coli* F^-^, lambda^-^, rph-1 | The Coli Genetic Stock Center at Yale |
| AR74 | *E. coli* ΔampG/ΔampD::kan | This study |
| *Plasmids* |  |  |
| pNU305 | pBR322 with ampRampC; tet+ | (1) |
| pACYC184 | p15; tet/cm | New England Biolabs |
| pACY-AR1 | Δtet; NdeI-HindIII linker;cm | This study |
| pACY-AR2 | BamHI & XhoI sites;cm | This study |
| pAC-TfAmpG | pACYC184 with Tanf_08365; cm | This study |
| pAC-lacTFgppX | pACYC184 derived expression vector with IPTG inducible (*lac* promoter) GppX-6xHis fusion | This study |
| pAC-lacTFgppΔHTH | pACYC184 derived expression vector with IPTG inducible (*lac* promoter) GppXΔHTH-  6xHis fusion | This study |
| pRS414  pRS-AmpG-promo | pBR322; amp  pRS414 with *T. forsythia* *ampG* promoter-*lacZ* fusion | (2)  This study |

amp, ampicillin resistance; kan, kanamycin resistance

1. Lindberg, F., Lindquist, S., and Normark, S. (1987). Inactivation of the ampD gene causes semiconstitutive overproduction of the inducible Citrobacter freundii beta-lactamase. *Journal of bacteriology* 169**,** 1923-1928.

2. Simons, R.W., Houman, F., and Kleckner, N. (1987). Improved single and multicopy lac-based cloning vectors for protein and operon fusions. *Gene* 53**,** 85-96.

**Table S2:** Primers used in this study. Introduced restriction sites are written in boldface.

| **Primer** | **Sequence (5’-3’)** |
| --- | --- |
| **P1 Transduction** | |
| AmpG Forward | AGCCATATTGCTGATCCTGG |
| AmpG Reverse | GCTAAGCCGCACAAAAGAAC |
| AmpD Forward | ACATGCTACTCTGAACCGGG |
| AmpD Reverse | CCGAAAGAACGCTTCAAGAC |
| **Construction of expression vectors** | |
| AmpGNde-F | GCGC**CATATG**AAAACGGGAAAGAAAACGAATGC |
| AmpGHind-R | GCGC**AAGCTT**ACAACGTGCACAAACACCAT |
| AmpGProEcoF | GCGCG**GAATTC**CTACAAGCCACTCTTTTCTCATGTATGTTAACCGCAGC |
| AmpGProRBam | CGCGC**GGATCC**ATTAATATGCATGCTTCTTCTTACACGGTGG |
| LacGppXF1 | GCTTCCGGCTCGTATAATGTGTGGAATTGTGAGCGGATAACAATTTCACACAGGAAAGATCTATGCTGACAATCGTACTTTATACCAACC |
| GppxXho1 | GCGC**CTCGAG**AAAGCCGATTCCTCTGCGATAATCCA |
| LacGppx-BamF2 | GCGCG**GGATCC**GGGCAGTGAGCGCAACGCAATTAATGTGAGTTACTCATTAGGCACCCCCAGGCTTTACACTTTATGCTTCCGGCTCGTATAATGTG |
| HTH-F | TGAATGGAAGCCGGCGGC |
| HTH-R | GCGC**CTCGAG**CGTGTTCACATCCAAGTGCCG |
| **RT-PCR** | |
| TF1059F | ATGTATCCTCGGTTTCCCCT |
| TF1061R | TCCGAATCGTTTCACTACCG |
| TF1061F | GGAAGACAATTCCGACGCT |
| TF1062R | ATGTCGTCGATGCGTCCT |
| TF1062F | ATCCGTCAGGTGATGGAAG |
| TF1063R | TTCGACAATCAGATGGAACG |
| TF1063F | AGCGGCAATACCCTTACGAT |
| TF1064R | AGCCCCAGCATATAAAAGCCG |
| TF1064F | GGATTCAGGAACACATCGGA |
| TF1065R | GTTTCCGTGTTTGCACCGTAG |
| TF1065F | TTATGCAGGAAATCGCGG |
| TFIR | GGAGAACCCGACACCTCC |
| **5’RACE** |  |
| TSS-Outer | GAGCAATTTGTATTGGCAAAAA |
| TSS-Inner | CACCATAAACACATTCTCTTATGC |

1 2 3 4


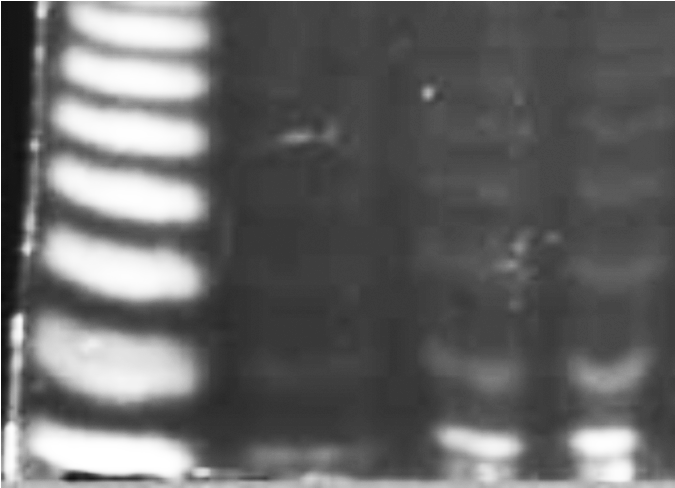


**Fig. S1.** Fluorophore-assisted carbohydrate electrophoresis (FACE) of peptidoglycan isolated from F. nucleatum. Matodextrin mixture ( and peptidoglycan samples were labeled with ANTS fluorophore and separated by electrophoresis using a 35% acrylamide gel. Lane 1; Maltodextrin mixture (dextrose equivalent 13-17); lanes 2-4, increasing concentrations of peptidoglycan (250 ng, 1 μg and 2.5 μg, respectively)

A


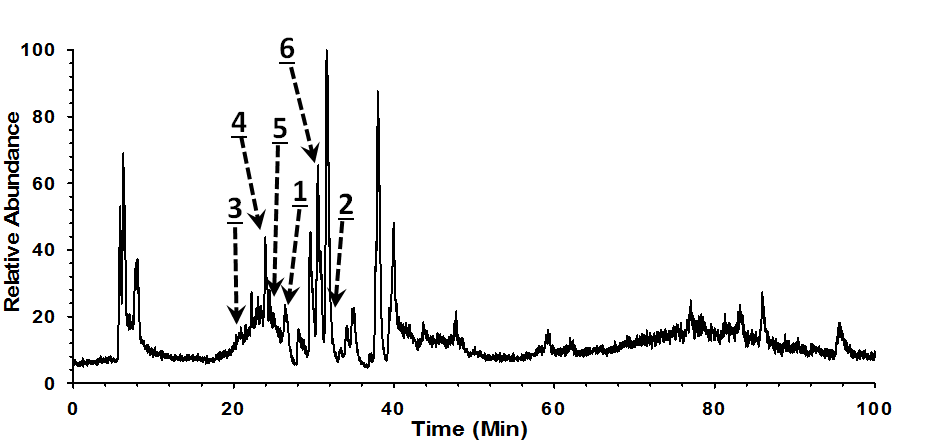


B

| **No.** | **Muropeptide** | **Retention time (Min)** | **Identification** | **Structure** |
| --- | --- | --- | --- | --- |
| 1 | Di | 26.58 | 697.03 [M+H] ^+^,  719.27 [M+Na] ^+^,  735.15 [M+K] ^+^, | GlcNAc-MurNAc-L-Ala-D-Glu |
| 2 | Di-Anhydro | 31.98 | 679.10 [M+H] ^+^,  701.35 [M+Na] ^+^, | GlcNAc-AnhMurNAc-L-Ala-D-Glu |
| 3 | Tri | 21.57 | 887.20 [M+H_2_O+H] ^+^ | GlcNAc-MurNAc-L-Ala-D-Glu-*m*-DAP |
| 4 | Tetra | 23.96 | 958.17 [M+H_2_O+H] ^+^ | GlcNAc-MurNAc-L-Ala-D-Glu-*m*-DAP-D-Ala |
| 5 | Penta | 25.03 | 1029.18 [M+H_2_O+H] ^+^ | GlcNAc-MurNAc-L-Ala-D-Glu-*m*-DAP-D-Ala-D-Ala |
| 6 | Tetra-Tetra | 30.58 | 940.17 [M+H_2_O+2H] ^2+^  949.45 [M+2H_2_O+2H] ^2+^  960.42 [M+2H_2_O+Na+H] ^2+^  968.58 [M+2H_2_O+K+H] ^2+^  1897.3 [M+2H_2_O+H] ^1+^ | (GlcNAc-MurNAc-L-Ala-D-Glu-*m*-DAP-D-Ala) — (GlcNAc-MurNAc-L-Ala-D-Glu-*m*-DAP-D-Ala) |

**Fig. S2.** Separation and identification of muropeptide by LC-MS: **A)** Chromatograph of Muropeptides derived from peptidoglycan of *F. nucleatum*, after mutanolysin digestion and separation on a C_18_-reversed phase column. The eluate was directly analyzed by mass spectrometry. The numbers indicate muropeptides for the table below with arrows pointing to the retention times on the chromatograph. **B)** Table shows muropeptides identified by their mass, as they elute out of the reversed phase column. Di, Tri, Tetra, Penta represents monomers where a disaccharide (GlcNAc-MurNAc) is linked to a di, tri, tetra and penta peptides respectively. Tetra-Tetra represents a dimer, where monomers are cross-linked at their peptide chains. Anhydro, represents 1,6-AnhydroMurNAc formation of which, in the peptidoglycan chain prevents further polymerization.


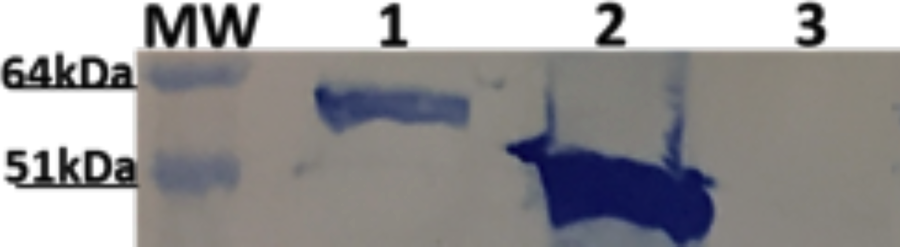


**Fig. S3.** Western immunoblotting of *E. coli* strains containing pAC-lacTfgppX and pAC-lacTfgppXΔHTH. After inducing protein expression with IPTG, western blot analysis of samples was performed using antibody against the His-tag to verify the expression of TfGppX or TfGppXΔHTH proteins tagged with 6xHis tag. A representative blot of three independent experiments is shown. MW: Molecular Weight (SeeBlue Plus 2 Invitrogen); 1, Full length GppX; 2, ΔHTH-GppX, and; 3, pACYC184 (negative control). Each lane received 2.5 μg total protein lysate.
